# Peripheral vascular responses to acetylcholine as a predictive tool for response to cholinesterase inhibitors in Alzheimer’s disease

**DOI:** 10.1186/s12883-019-1316-4

**Published:** 2019-05-03

**Authors:** Peter J. Connelly, Fiona Adams, Ziad I. Tayar, Faisel Khan

**Affiliations:** 1 Murray Royal Hospital and University of Dundee, Muirhall Road, Perth, PH2 7BH UK; 2 Division of Molecular and Clinical Medicine, Ninewells Hospital and Medical School, University of Dundee, Dundee, DD1 9SY UK; 3 Kingsway Care Centre, Dundee, UK

**Keywords:** Cholinesterase, Acetylcholine, blood flow, Laser Doppler, vascular response, Prediction

## Abstract

**Background:**

Cholinesterase inhibitors remain the first line therapy for people with mild to moderate Alzheimer’s disease (AD). Response is modest and difficult to predict from pre-treatment characteristics. We hypothesise that skin vascular response to iontophoresis of acetylcholine, which is partly determined by the level of cholinesterase activity, may be a pre-treatment measure that could predict response to therapy.

**Methods:**

Twenty-four people with probable AD underwent iontophoresis of acetylcholine to the volar surface of the forearm skin prior to treatment with a cholinesterase inhibitor. The peak skin vascular response and the resolution to baseline levels were measured using laser Doppler perfusion imaging. Response to treatment was assessed after 6 months using criteria from the National Institute for Health and Care Excellence (NICE) and iontophoresis with acetylcholine was repeated. Blindness between clinical and laboratory assessments was maintained.

**Results:**

Fourteen out of twenty-four people responded to treatment using NICE criteria. By comparison to non-responders, responders to treatment had a faster resolution to baseline from acetylcholine-induced vasodilation prior to treatment, which slowed with treatment. In this pilot study there was a high level of accuracy in the classification of response using this variable. No baseline cognitive or functional measures discriminated end-point responders from non-responders.

**Conclusion:**

Cholinesterase inhibitors are well tolerated but the number of people with adverse effects would be reduced if it was possible to predict response. The role of vasodilator response to acetylcholine and recovery as a potential biomarker for efficacy of treatment should now be evaluated and may possibly be of relevance in stratifying samples for interventional studies in AD and other forms of dementia. We feel that a more definitive study is now justified.

## Background

Cholinesterase inhibitors (Donepezil, Rivastigmine and Galantamine) are the first line therapy for people with mild to moderate Alzheimer’s disease (AD). Beneficial effects have been shown on intellectual function, day-to-day abilities and social behaviour for all three drugs [1]. However, only approximately 40% of people who take these drugs have a good response [1]. It remains extremely difficult to predict in advance who might respond though an association between pre-treatment processing speed and response has been demonstrated [2]. Improved regional cerebral blood flow (rCBF) has also been correlated with clinical improvement [3–5], though there is dispute over the baseline characteristics of rCBF which might be associated with response to treatment [6, 7].

Altered skin microvascular reactivity has been described in AD [8, 9] suggesting that skin microvascular reactivity might be useful in the diagnosis of AD. A reduced skin vasodilator response to iontophoresis of acetylcholine has been described in untreated patients with mild AD compared with control subjects [8], while a significantly enhanced response in patients treated with Donepezil by comparison to untreated AD patients has also been found [10]. Our group have previously shown that the duration of the skin vascular response to iontophoresis of acetylcholine, in particular the recovery to baseline, is partly determined by the level of cholinesterase activity [11, 12]. AD is known to be associated with lower levels of acetylcholine. Consequently, less acetylcholine will be available for hydrolysis and circulating acetylcholinesterase (AChE) levels will be potentially higher in people with AD. We therefore hypothesised that a person with a relatively fast acetylcholine recovery response could be expected to have high cholinesterase activity and therefore might be a good candidate for response to cholinesterase inhibitor treatment.

This study attempts to identify the characteristic pattern of change in peripheral blood flow following exposure to iontophoresis of a standard dose of acetylcholine and assess its association with response to cholinesterase inhibitor therapy in people with AD. In particular, we sought to determine whether there a difference over 6 months in the magnitude of change in blood flow between people with AD classed as “responders” or “non-responders” to cholinesterase inhibitor therapy and whether blood flow responses prior to treatment predict response to cholinesterase inhibitors in patients with AD. The identification of a marker for ‘good responders’ to cholinesterase inhibition would improve the cost-benefit of this class of drug as well as reducing risks in those unlikely to respond to treatment.

## Methods

The study was of pragmatic design and with the main inclusion criterion being diagnosis of probable AD by a Consultant Psychiatrist or Senior Trainee using ICD-10 criteria as is standard practice in the local Memory Service in Tayside after assessment of the chronology of key symptoms including intellectual, functional and social change and, where appropriate, neuroimaging. The decision to prescribe a cholinesterase inhibitor, and the choice of drug, resided with the clinician in charge of the patient’s clinical care. Participants were invited to enrol in the study at the point of diagnosis. Only those judged to have capacity to consent were included. All participants gave written informed consent to the study, which was approved by the East of Scotland Research Ethics Service (REC reference 11/AL/0259). Since all patients who are suitable for cholinesterase inhibitor therapy are commenced on those drugs it was not possible to include a matched control group.

For the purposes of this study, participants who consented to participate underwent assessment using the Mini-Mental State Examination (MMSE) [13], the Nurses’ Observation Scale for Geriatric patients (NOSGER) [14] and a version of the Digit Symbol Substitution Test (DSST) [15]. The Instrumental Activities of Daily Living and Social Behaviour sub-scales of the NOSGER were extracted from the full score as these have been used in other local studies as an index of change involving with no memory questions. The study was approved by the East of Scotland Research Ethics Committee (ref 11/AL/0259) and conducted according to the principles of the Declaration of Helsinki. Recruitment ran from October 2011 to May 2014.

Since the study was designed to reflect actual clinical practice in the use of cholinesterase inhibitors, no exclusions were made on the use of concurrent drug therapy, smoking status, hypertension or diabetes. Similarly there was no set range for Mini Mental State Examination (MMSE) scores at baseline; we felt this was justified because an association between MMSE scores and vasodilator response was not found in a study by Maltz et al. [10] in similar patients. Patients were excluded if they had medical conditions known to interfere with acetylcholine metabolism or were likely to react adversely to skin stimulation. Additionally, we excluded patients if they were taking medication whose primary action was anti-cholinergic and was being used for a medical condition in which anti-cholinergic action was the desired intervention e.g. oxybutynin for irritable bladder. Smoking and caffeine intake were prohibited on the day of skin testing until all tests had been completed.

Prior to commencing treatment with a cholinesterase inhibitor (usually within 10 working days) patients had baseline skin blood flow responses to iontophoresis of acetylcholine and sodium nitroprusside performed by an experienced Research Fellow (FA) or under her supervision. All tests were conducted in the same laboratory setting within Ninewells Hospital, Dundee, UK. All assessments were undertaken in the morning to control for the diurnal variation of vascular responses [16]. Blindness between the results of any cognitive, functional or global assessments and the results of skin flow measures was maintained throughout the study.

### Iontophoresis of vasoactive chemicals

Experiments were conducted in a temperature-controlled room (22-23 °C). Following a 20-min equilibration period, baseline skin perfusion was measured for 2 min. Skin blood flow responses to iontophoresis of acetylcholine and sodium nitroprusside were measured on the volar aspect of the forearm using laser Doppler perfusion imaging (moorLDI, Moor Instruments Ltd., Devon, UK) according to our previously described protocol [12, 17]. Scans at 30 s intervals were used to build colour-coded image representing skin perfusion in two dimensions. From these images, a relative measure of skin blood flow was determined (termed laser Doppler flux) and expressed in perfusion units (PU). While the recovery of the acetylcholine response is partly determined by levels of cholinesterase, the peak response is governed by the endothelium-dependent production of nitric oxide. Therefore, as a control, the skin blood flow response and recovery to iontophoresis of sodium nitroprusside were determined since these results are determined by the endothelium-independent production of nitric oxide.

Iontophoresis was used to transport 1% acetylcholine (Miochol-E, Novartis, Surrey, UK) for 80 s using a 0.1 mA anodal current (delivering a dose of 8 m-Coulombs (mC)) across the skin within the area of the iontophoresis chamber (Moor Instruments Ltd., Devon, UK), which consisted of a Perspex ring of internal diameter 20 mm. After determining the peak blood flow response to acetylcholine, the subsequent recovery of blood flow from this level back to baseline was recorded for up to 30 min to allow the rate of decay to be established. Following this, 1% sodium nitroprusside (Rottapharm, Barcelona, Spain) was iontophoresed at an adjacent site for 80 s using a 0.1 mA cathodal current. To determine the decay response, the time taken for the blood flow to return to 50% of the peak value for acetylcholine was calculated.

After the initial measurements of blood flow response to iontophoresis of acetylcholine and sodium nitroprusside patients took the first dose of cholinesterase inhibitor medication. All three drugs (Donepezil, Rivastigmine and Galantamine) were used in the study. At 6 months, participants were assessed clinically by the Consultant psychiatrist or senior psychiatric trainee responsible for the clinical care of the patient to determine whether they were considered to have responded to the treatment or not, with classification as ‘responder’ or ‘non-responder’ based on the change in MMSE, NOSGER sub-scales, DSST and a global assessment of change including carer/family views (see Table 1) as recommended by NICE [18]. Blood flow responses were re-measured as described above.

**Table 1:** Baseline clinical characteristic of AD patients. Values are means ± SE

	All (*n* = 24)	Non-responder (*n* = 10)	Responder (*n* = 14)
Age (years)	77.6 ± 1.2	78.8 ± 2.0	76.6 ± 1.6
Male/female (No.)	12/13	3/7	9/5
Height (m)	1.62 ± 0.02	1.59 ± 0.03	1.65 ± 0.02
Weight (Kg)	72.3 ± 2.7	77.6 ± 4.9	69.1 ± 3.5
Body mass index (Kg/m^2^)	27.3 ± 1.0	30.5 ± 1.6	25.2 ± 1.0*
Systolic blood pressure (mmHg)	149 ± 4	150 ± 5	145 ± 6
Diastolic blood pressure (mmHg)	76 ± 2	80.1 ± 3	74.4 ± 3
Heart rate (beats/min)	66 ± 2	72 ± 3	64 ± 3

* *P* < 0.01

### Statistical analysis

Differences in vascular responses to acetylcholine and sodium nitroprusside between good and poor responders was analysed using two way analysis of variance and unpaired t-tests. Within group differences were analysed using paired t-tests. Correlations were performed using Pearson’s correlation coefficient. *P* < 0.05 was chosen as the level of significance. All comparisons were undertaken using SPSS Version 22. Analysis of cognitive and functional scales or blood flow data was performed by individuals who had remained blind to the results from the other domains throughout the study.

## Results

Twenty-six patients were enrolled and twenty-four completed the follow-up visit at 6 months. Data is therefore presented only for those participants who completed both visits. The main reason for two participants withdrawing from the study was a reluctance to travel to the laboratory site. No study procedure-related adverse events were recorded. Baseline clinical characteristics of the patients are shown in Table 1.

Table 2 shows the data in all patients for clinical responses and vascular responses at baseline and following 6 months of treatment. No significant differences were observed for any of the clinical assessments or vascular parameters measured at baseline versus 6-months’ follow-up, although there was a trend for an increase in peak responses to acetylcholine (*P* = 0.08) and sodium nitroprusside (*P* = 0.08) at 6 months.

**Table 2:** Changes in clinical assessments and vascular responses (in perfusion units) to acetylcholine and sodium nitroprusside in all patients (*n* = 24), and the subsequent time course of decay in acetylcholine response to 50% of the peak value at baseline and following 6 months of treatment. Values are means ± SE

	Baseline	6 months
Clinical parameters		
MMSE	24.0 ± 0.6	24.7 ± 0.6
NOSGER IADL/SB	37.8 ± 1.6	37.5 ± 1.7
NOSGER Total	118.9 ± 3.7	118.7 ± 3.3
DSST	21.4 ± 2.3	21.9 ± 2.1
Vascular responses		
Basal perfusion (PU)	40 ± 11	47 ± 12
Peak acetylcholine response (PU)	334 ± 40	389 ± 31
Peak sodium nitroprusside response (PU)	274 ± 34	337 ± 33
Acetylcholine 50% decay (secs)	318 ± 41	321 ± 32

*MMSE* Mini Mental State Exam

*NOSGER* Nurses Observation Scale for Geriatric Patients IADL/SB combined score (max 50, higher scores are better)

*NOSGER Total* Nurses Observation Scale for Geriatric Patients Total Score (max 150, higher scores are better)

*DSST* Digit Symbol Substitution Test over 90 s

### Response to treatment

After 6 months’ treatment, 14/24 (58.33%) were classified as “responders” to cholinesterase therapy. There were no significant differences in the basic clinical characteristics between responders and non-responders other than body mass index. (Table 1).

Table 3 shows the data for responders and non-responders for clinical and vascular responses at baseline and following 6 months of treatment. No significant differences were observed for any of the vascular parameters measured at baseline versus 6-months’ in non-responders.

**Table 3:** Changes in clinical assessments and vascular responses (in perfusion units) to acetylcholine and sodium nitroprusside in responders and non-responders, and the subsequent time course of decay in acetylcholine response to 50% of the peak value at baseline and following 6 months of treatment. Values are means ± SE

	Non responder		Responder	
	Baseline	6 months	Baseline	6 months
Clinical parameters				
MMSE	23.4 ± 1.0	23.0 ± 0.9	24.4 ± 0.6	25.9 ± 0.6
NOSGER IADL/SB	39.0 ± 2.2	35.9 ± 2.6*	36.8 ± 2.4	38.8 ± 2.2
NOSGER Total	121.8 ± 4.8	116.2 ± 4.7	116.5 ± 5.5	120.7 ± 4.7
DSST	16.6 ± 3.7	14.5 ± 3.8*	22.3 ± 2.5	25.1 ± 1.8
Vascular responses				
Basal perfusion (PU)	49 ± 12	51 ± 13	41 ± 10	43 ± 11
Peak acetylcholine response (PU)	345 ± 77	361 ± 49	326 ± 43	409 ± 43**
Peak sodium nitroprusside response (PU)	288 ± 68	296 ± 45	265 ± 36	366 ± 50***
Acetylcholine 50% decay (secs)	400 ± 60	321 ± 32	230 ± 38¥	332 ± 42*

* *P* < 0.05; ** *P* < 0.01; *** *P* < 0.005, comparing values at baseline versus 6 months within the two groups

¥ *P* < 0.05 comparing baseline acetylcholine 50% decay between non-responders and responders

Non-responders showed a decline in values for all four clinical variables whereas responders showed improvement in all four, although only IADL/SB and DSST scores among the non-responders were significantly different from baseline within the groups. Analysis of covariance showed significant differences between the change in scores for all variables from baseline to 6-months follow-up between the two groups after controlling for baseline scores (MMSE *P* = 0.013, NOSGER IADL/SB *P* = 0.031, NOSGER Total *P* = 0.013, DSST *P* = 0.005).

Within the responder group, there were significant increases in the peak acetylcholine response (*P* < 0.01) and peak sodium nitroprusside response (*P* < 0.005) between responses at baseline versus follow-up at 6 months, and also a significant increase in the time course of acetylcholine 50% decay (*P* < 0.05) indicative of a slower decay response. The change in peak acetylcholine response, peak sodium nitroprusside response and acetylcholine 50% decay from baseline to 6-month follow-up was significantly different between non-responders and responders after adjusting for baseline values, BMI and gender (*P* < 0.002, *P* < 0.01, and *P* < 0.001, respectively). Within the non-responder group, there were no significant differences in peak acetylcholine response, peak sodium nitroprusside response, or in the time course of acetylcholine 50% decay before and after treatment.

Comparing responses between non-responders and responders at baseline and at follow-up at 6 months showed that acetylcholine 50% decay was significantly faster in responders (230 ± 38 s versus 400 ± 60 s, *P* < 0.05) indicating a faster decay in patients who were subsequently classified as responders. No other differences were observed between the two groups at baseline or at 6 months follow-up.

There were no significant correlations between the baseline variable values in Table 1 and vascular responses to acetylcholine and sodium nitroprusside or in the time course of acetylcholine 50% decay in all patients at baseline and 6-months follow-up or within the non-responder and responders groups at any time point. However, there were negative correlations between baseline peak and decay values for acetylcholine and change scores over time for MMSE, NOSGER IADL/SB, NOSGER Total and DSST, which were significant for NOSGER IADL/SB in the total group (*r* = − 0.575, *P* = 0.006 and *r* = − 0.435, *P* = 0.049 for peak acetylcholine and decay, respectively) and for both NOSGER IADL/SB (*r* = − 0.817, *P* = 0.001 for peak acetylcholine) and NOSGER Total (*r* = − 0.704 *P* = 0.016 for peak acetylcholine response) in the responders. No comparisons were significant in non-responders.

Receiver operating characteristic curve analysis between non-responders and responders versus baseline acetylcholine peak or decay revealed an area under curve value of 0.583 for peak and 0.800 for decay with a cut-point for decay of 242 s showing sensitivity of 0.90 and specificity of 0.77 for categorisation of response group. 10/14 responders and 1/10 non-responders had a baseline decay time ≤ 242 s. Bivariate logistic regression around this cut-point showed that 79.2% of patients were correctly categorised (positive predictive value = 71.4%, negative predictive value 90%) (Wald statistic = 6.64, df = 1, *P* = 0.010). Odds ratio was 22.50 (95% CI 2.11–240.48).

## Discussion

The findings from this study show that the clinical response to cholinesterase inhibitors over a 6 month period is modest as expected, although there are different patterns in responders versus non-responders. No baseline cognitive or functional variable was able to discriminate end-point responders from non-responders at a statistically significant level. There was also no relationship between perfusion parameters and severity of cognitive or functional impairment at baseline. By contrast, baseline decay in recovery from vasodilation associated with acetylcholine discriminated the response groups with a high degree of accuracy. There were negative correlations between baseline decay and the change in each clinical variable, suggesting the possibility that the degree of inhibition of AChE may be related to response as previously reported with respect to CSF [19], which is considerable more invasive to measure. As a rapidly measured dynamic variable, our test may also prove more useful that measuring red cell cholinesterase inhibition, which has been shown to be an unreliable marker for the activity of cholinesterase inhibitors [20].

Recovery of the vasodilator response to iontophoresis of acetylcholine is probably dependent upon various factors, one of which will be the vascular expression of cholinesterase. We have previously shown that high cholinesterase activity is normally associated with a quicker recovery in the acetylcholine-induced blood flow response [12], while a prolongation of the decay of acetylcholine-stimulated hyperaemia in response to topical edrophonium administration in the forearm of normal human subjects has also been shown [21]. The prolongation of decay of the iontophoretic response to acetylcholine through an effect on inhibition of cholinesterase via malathion lends further support to the link between skin response and vascular cholinesterase activity [22].

The fact that the peak acetylcholine response following a single dose application is not statistically different between non-responders and responders at baseline suggests that muscarinic receptor density on the endothelium is comparable and that there were no significant differences in endothelial function. Additionally, the finding that responses to sodium nitroprusside were also similar between non-responders and responders at baseline implies that general vascular function was comparable in the two groups.

Although BMI was significantly greater in non-responders than responders at baseline, we did not find any correlation between this and acetylcholine decay and cannot comment on what influence this might have on the response to treatment. However, an important point to note is that the purpose of our study was not to determine what factors (e.g. BMI, blood pressure, gender) influence outcome to treatment, but rather to see whether we could find a biomarker indicative of response to treatment based on examining the peripheral vascular response to iontophoresis of acetylcholine.

We appreciate that additional factors that were not assessed in this study could change the cholinergic status and alter the capacity to hydrolyse acetylcholine and thus the responses found in our test [23, 24], but possibly also in treatment. We recognise that many drugs have anticholinergic actions of differing potency [25–27] and that some of these were used for concomitant medical disorders, but given the sample size in our pilot study it is unrealistic to use potency as a variable in our analyses. In our larger planned study we will address this issue more closely. Participants who had previously been treated with cholinesterase inhibitors or who had medical conditions known to interfere with acetylcholine metabolism were excluded.

We are also aware that a peripheral test can only be a proxy for central activity and a weakness of our pilot study is that it did not allow for detailed examination of the mechanism which might affect response including potentially important genomic findings in single nucleotide polymorphisms (SNPs) in both the acetyl- and butyrylcholinesterase (AChE and BChE) genes which may have a strong influence on the responses found in our test [24] but possibly also in treatment. However, the strength of our findings is that they are generated by a minimally-invasive test which can be performed fairly quickly in a clinic setting and potentially provide a rapid pragmatic guide to clinicians and patients about likelihood of response to cholinesterase inhibitors. It is highly likely that no single variable will prove to be a fully predictive measure of response but detailed mechanistic evaluation can be explored as part of a larger follow-up study in preparation.

Following 6-months’ of treatment, there were no significant changes in any of the vascular function parameters in the non-responders which was in contrast with the responders who showed significant slowing in the acetylcholine decay at 6-months compared with baseline and also a significant improvement in vascular responses to acetylcholine and sodium nitroprusside. We believe that the slower decay in responders is related to a decrease in cholinesterase activity due to inhibition by treatment. The reasons for the improvement in the peak response to both acetylcholine and sodium nitroprusside is less clear but may be related to an improvement in endothelial function in the case of acetylcholine [8], and, for sodium nitroprusside, due to an improvement in release of acetylcholine due to flow-mediated dilatation following cholinesterase inhibition [28].

The observed increase in skin blood flow response to acetylcholine and sodium nitroprusside might also reflect improvements in cerebral perfusion and thus contribute to the positive response to treatment. Vascular responses in the skin are thought to reflect generalised vascular function [29]. We have previously shown that skin vascular responses to iontophoresis of acetylcholine and sodium nitroprusside in the forearm correlate significantly with responses in the coronary circulation [30], and thus it is plausible that similar improvements could be present in cerebral vessels and thus explain the findings on SPECT seen in earlier studies [3–5]. In this way, improvements in cerebral blood flow could contribute to slowing of neural degeneration.

## Conclusion

Though we accept that in this pilot study numbers were small and that replication in a larger study is required, these results are encouraging and support our hypothesis that levels of cholinesterase are higher than in non-responders and thus treatment is more likely to be of benefit in people with faster decay of vasodilator response. The implications of our findings, if replicated in a larger study which controls for possible vascular confounders and factors thought to interact with cholinesterase activity (such as genomic differences and blood AChE and BChE activities), are that it may be possible to predict response to cholinesterase inhibitors by using a brief non-invasive test prior to treatment. By contrast to studies relating clinical response to changes in rCBF [3–5], our study identifies a possible baseline variable unrelated to severity of cognitive or functional impairment, rather than measuring change in pre-defined groups.

In addition to possible longer-term benefits for those who respond to treatment, avoiding prescription in potential non-responders prevents exposure to potential side-effects of these drugs and allows appropriate care plans to be drawn up which concentrate on non-pharmacological interventions. The role of vasodilator response as a potential biomarker for treatment should now be evaluated and may possibly be of relevance in stratifying samples for interventional studies in AD and other forms of dementia. We feel that a more definitive study is now justified.

## Abbreviations

AChE: Acetylcholinesterase
AD: Alzheimer’s disease
BChE: Butyrylcholinesterase
DSST: Digit Symbol Substitution Test
IAD: Instrumental activities of daily living
mC: milli-Coulombs
MMSE: Mini-Mental State Examination
NICE: National Institute for Health and Care Excellence
NOSGER: Nurses’ Observation Scale for Geriatric patients
PU: Perfusion units
rCBF: Regional cerebral blood flow
SB: Social behaviour
